# Author Correction: Stabilizing indium sulfide for CO_2_ electroreduction to formate at high rate by zinc incorporation

**DOI:** 10.1038/s41467-021-26430-5

**Published:** 2021-10-15

**Authors:** Li-Ping Chi, Zhuang-Zhuang Niu, Xiao-Long Zhang, Peng-Peng Yang, Jie Liao, Fei-Yue Gao, Zhi-Zheng Wu, Kai-Bin Tang, Min-Rui Gao

**Affiliations:** Division of Nanomaterials & Chemistry, Hefei National Laboratory for Physical Sciences at the Microscale, University of Science and Technology of China, Hefei, 230026 China

**Keywords:** Electrocatalysis, Materials chemistry, Electrocatalysis

Correction to: *Nature Communications* 10.1038/s41467-021-26124-y, published online 5 October 2021.

In this article the affiliation details for all authors were incorrectly given as ‘Division of Nanomaterials & Chemistry, Hefei National Laboratory for Physical Sciences at the Microscale, Hefei, China’ but should have been ‘Division of Nanomaterials & Chemistry, Hefei National Laboratory for Physical Sciences at the Microscale, University of Science and Technology of China, Hefei 230026, China’. The original article has been corrected.

