# Supplementary material for: Stabilizing indium sulfide for CO2 electroreduction to formate at high rate by zinc incorporation
Source: Nat Commun. 2021 Oct 5;12:5835. doi: 10.1038/s41467-021-26124-y (PMC8492718; doi:10.1038/s41467-021-26124-y)
Supplement: Supplementary file 1 — Supplementary Information [file 41467_2021_26124_MOESM1_ESM.pdf]

Supplementary Information for

# Stabilizing indium sulfide for $CO_2$ electroreduction to formate at high rate by zinc incorporation

Chi et al.

## Supplementary Figures

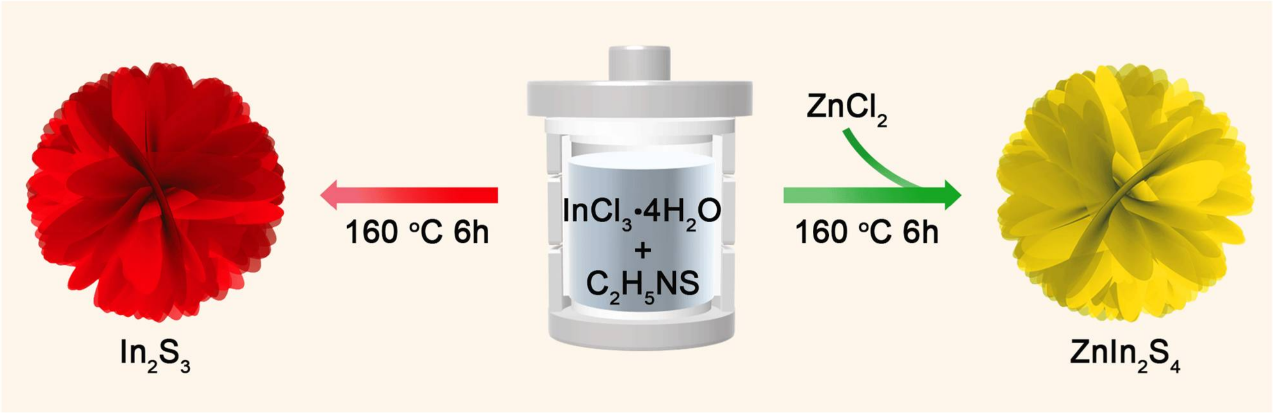


**Supplementary Figure 1. Schematic of the synthesis of $In_2S_3$ and $ZnIn_2S_4$.** The synthesis of the two samples are almost identical, giving rise to similar morphology and size. The only difference is the addition of $ZnCl_2$ when synthesizing the $ZnIn_2S_4$ sample.

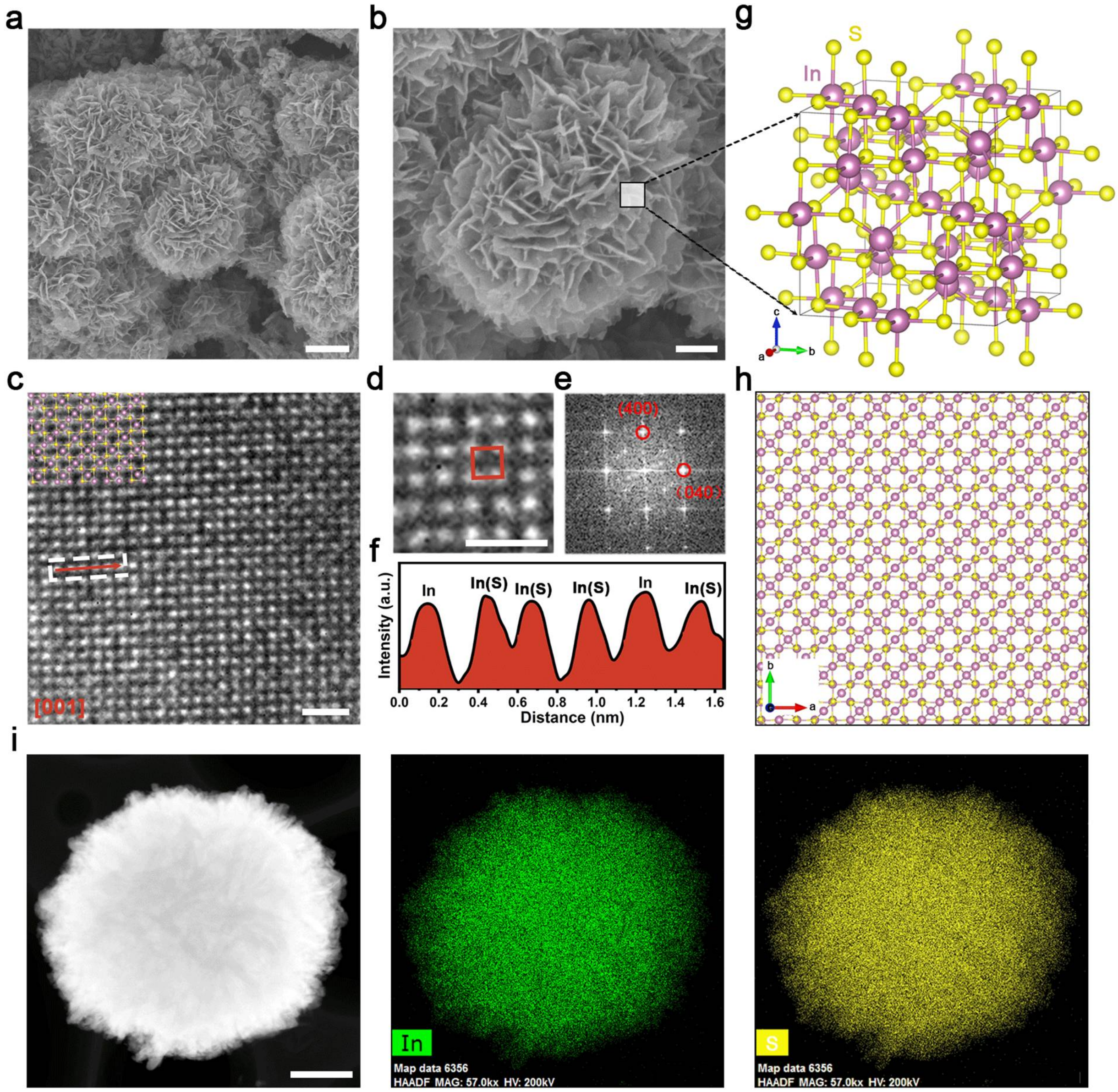


**Supplementary Figure 2. Physical characterization of the $In_2S_3$. a, b,** SEM images. **c, d,** Atomic-resolution Z-contrast images along [001] zone axis. Inset in **c** gives corresponding atomic arrangement. **e,** The corresponding FFT pattern of **c**. **f,** The line intensity profile acquired along the red arrow of **c. g, h,** Atomic model of $In_2S_3$ depicts that part of In atoms are located at the centres of the square. **i,** STEM-EDX elemental mapping of $In_2S_3$, showing clearly the homogeneous distribution of In (green) and S (yellow), respectively. Scale bars, **a,** 500 nm, **b,** 200 nm, **c, d,** 1 nm and **i,** 300 nm.

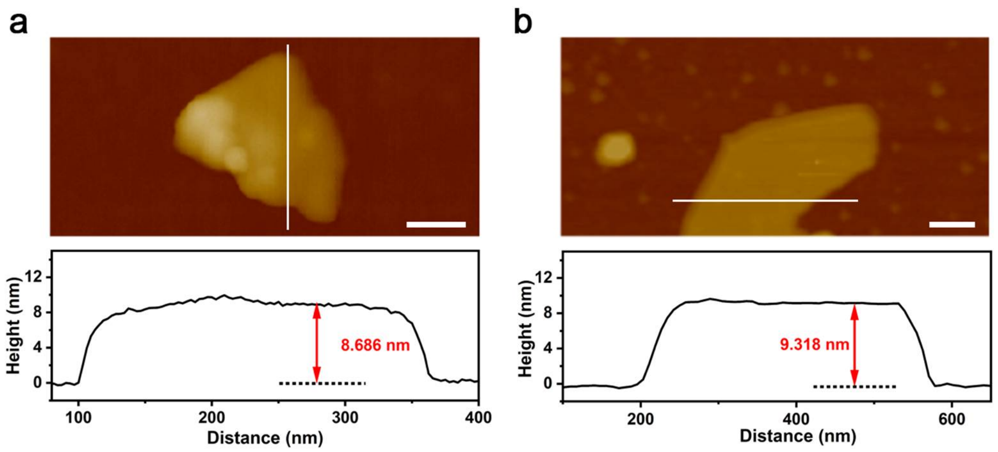


**Supplementary Figure 3. Thickness measurement.** The AFM images and corresponding height profiles of $ZnIn_2S_4$ (**a**) and $In_2S_3$ (**b**). Scale bars, 100 nm (**a**) and 200 nm (**b**).

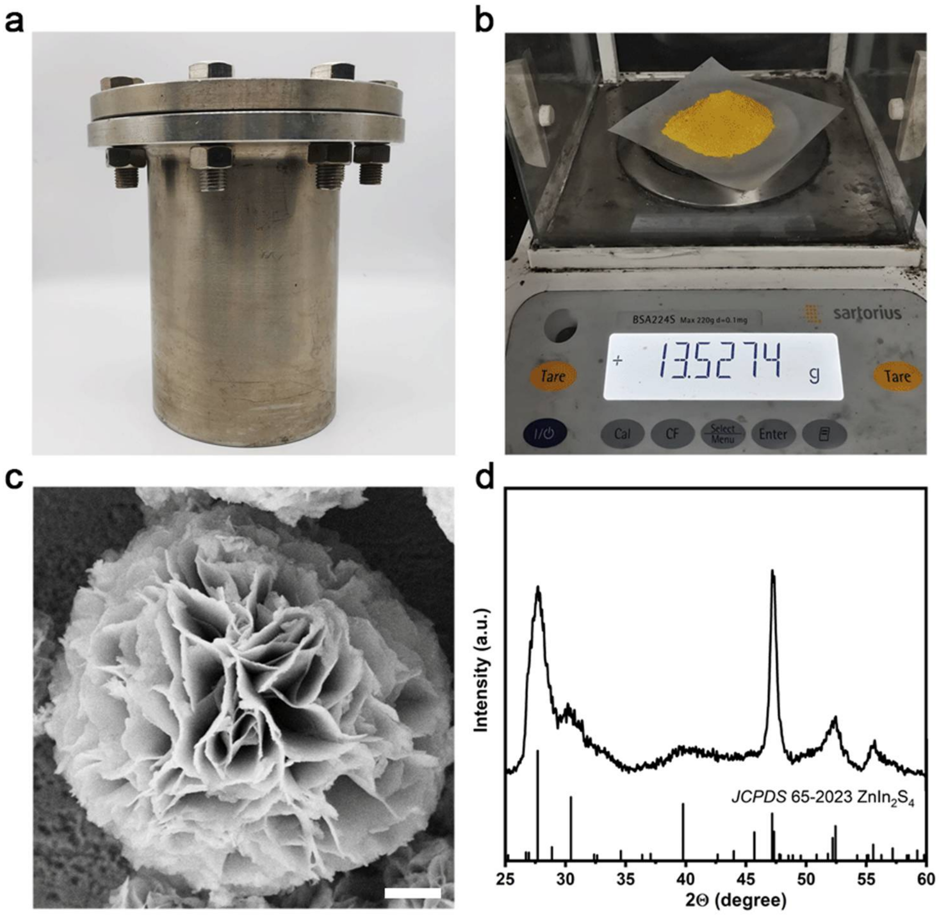


**Supplementary Figure 4. One-batch synthesis of 13.5 g $ZnIn_2S_4$. a,** The photograph of hydrothermal reactor with a volume of 1.5 L. **b,** $ZnIn_2S_4$ products on a balance scale. The SEM image (**c**) and XRD pattern (**d**) verify the successful high-yield synthesis of $ZnIn_2S_4$ catalyst, implying a potentially large-scale use. Scale bar, 1 μm.

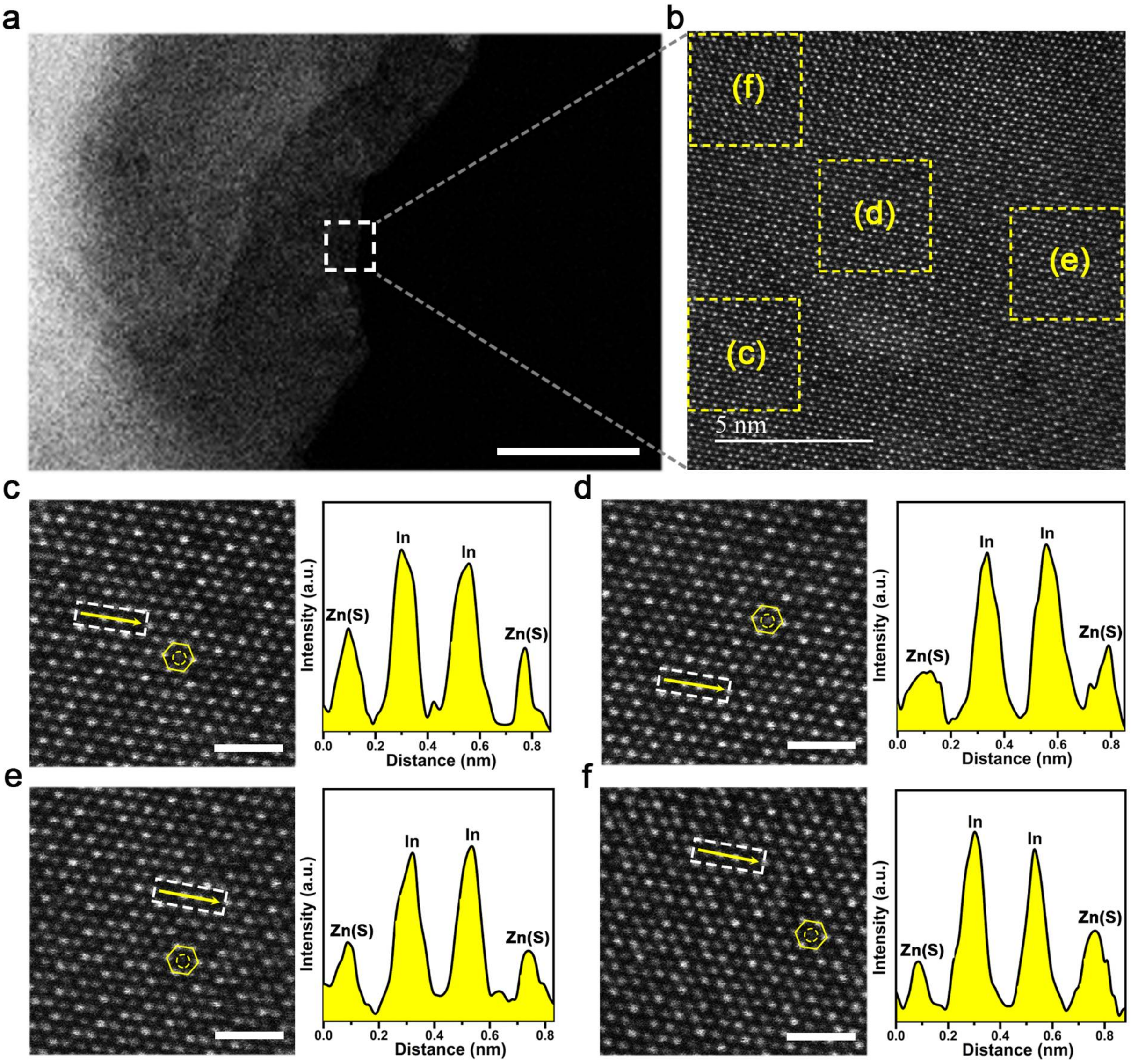


**Supplementary Figure 5. Atomic level characterization of $ZnIn_2S_4$. a,** HAADF-STEM image. **b,** Atomic-resolution Z-contrast image taken on the edge of $ZnIn_2S_4$ nanosheets in **a. c-f,** Left, enlarged atomic-resolution Z-contrast images corresponding to the regions highlighted with yellow boxes in **b,** respectively; Right, the line intensity profile acquired along the yellow arrows, respectively. Scale bars, **a,** 50 nm, **b,** 5 nm and **c-f,** 1 nm.

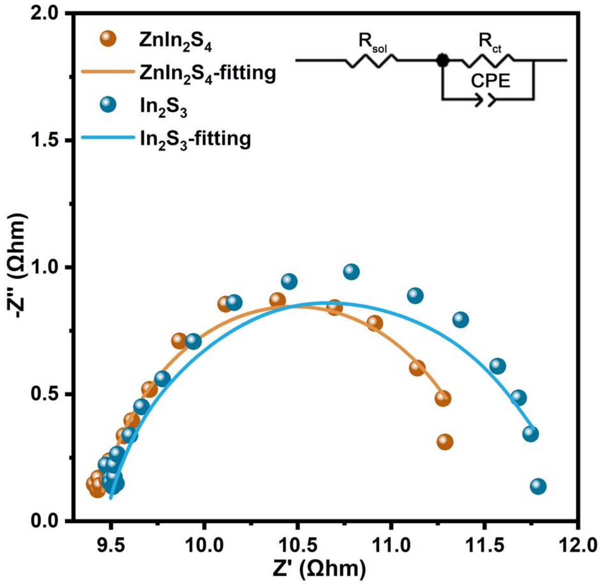


**Supplementary Figure 6. EIS Nyquist plots.** The EIS measurement was performed at -0.6 V *vs* RHE in $CO_2$-fed 1 M $KHCO_3$ over a frequency range from 100 KHz to 10 mHz at the amplitude of the sinusoidal voltage of 5 mV. The solution resistance ($R_{sol}$) is ~9.5 ohms for $ZnIn_2S_4$ and $In_2S_3$. The result shows a lower charge transfer resistance ($R_{ct}$) of $ZnIn_2S_4$ (~1.6 ohms) compared to $In_2S_3$ (~2.0 ohms), suggesting superior charge-transfer kinetics of $ZnIn_2S_4$. The fitting curves were obtained through the R(QR) equivalent circuit, which match well with the EIS datas.

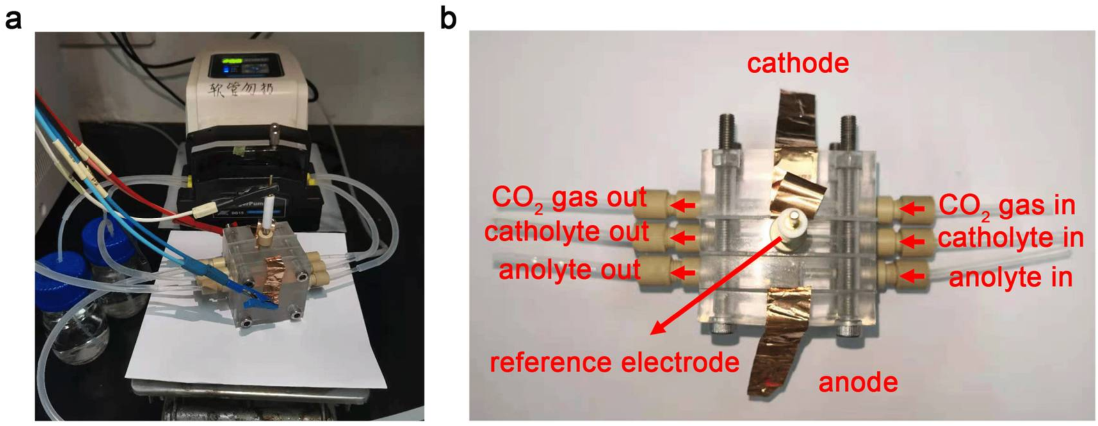


**Supplementary Figure 7. Photographs of the flow cell setup.** (**a**) Photograph of the flow cell setup during $CO_2$ electrolysis. (**b**) Photograph with notes that give detailed descriptions about the structure of the flow cell setup. The flow cell setup consist of two chambers for liquid electrolytes (catholyte and anolyte) and one gas chamber for $CO_2$ gas. During $CO_2$ electrolysis, the catholyte and anolyte were circulated between the chamber of the reactor and the liquid storage tank through the silicone tube. $CO_2$ gas flowed into the gas chamber at the back side of the GDE (1×1 $cm^2$), and then flowed to the on-line GC through the silicone tube. The cation exchange membrane (Nafion$^{TM}$ 117) was used to avoid the crossover issue of the negative formate. An Ag/AgCl (saturated KCl) reference electrode was placed in the cathode chamber to obtain the potential of the cathode. The cathode and anode (nickel foam) were connected to an external electrochemical workstation with the conductive copper tape, respectively.

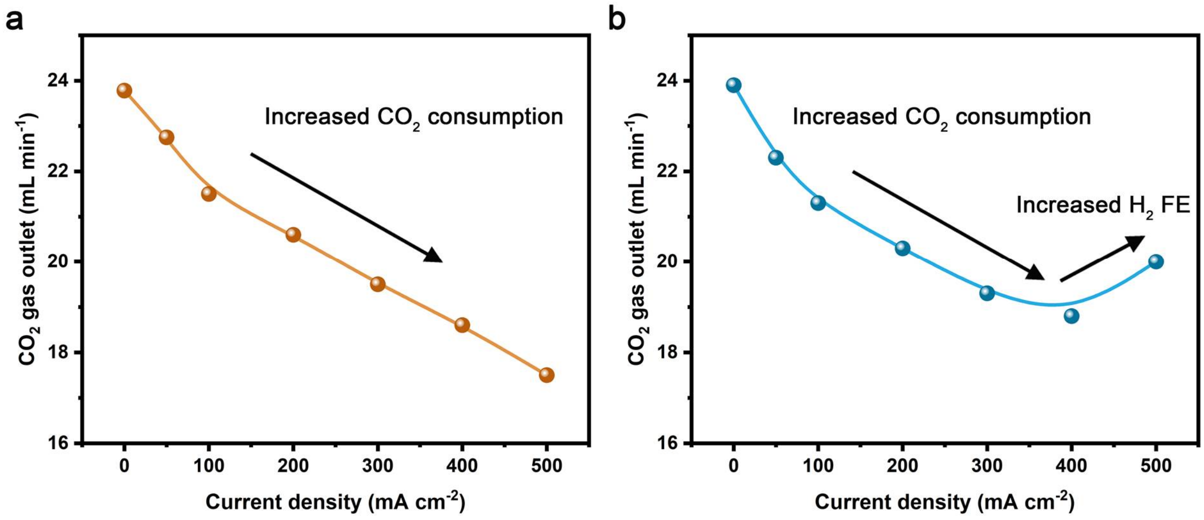


**Supplementary Figure 8. Measurement of $CO_2$ outlet gas flow rate.** A mass flowmeter was used to carefully monitor the $CO_2$ outlet gas flow on the $ZnIn_2S_4$ (**a**) and $In_2S_3$ (**b**) under various current densities for accurate product analysis. The $CO_2$ gas inlet flow rate was maintained at 24 mL min$^{-1}$ through the mass flow controller. The results show that as the current density increases, the outlet flow rate of $ZnIn_2S_4$ decreases gradually accompanied by increased $CO_2$ consumption. By contrast, the outlet flow rate of $In_2S_3$ rebounded at 500 mA cm$^{-2}$, ascribed to the increase in $H_2$ product.

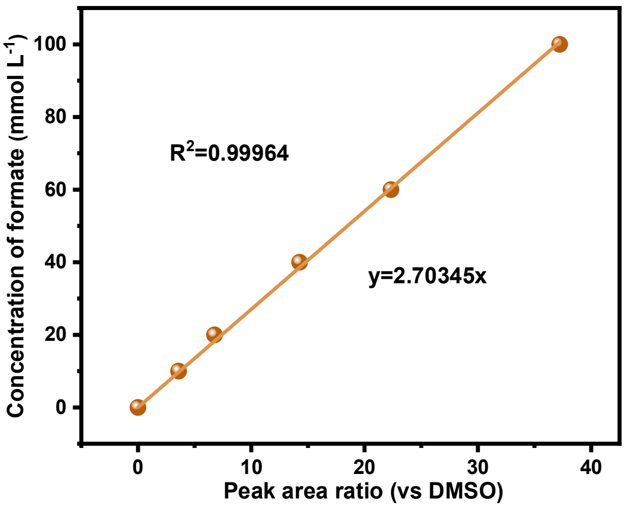


**Supplementary Figure 9. Method for quantification of formate concentration.** The concentration of formate is quantified through the standard curve of formate by plotting the formate concentration with respect to the formate/DMSO NMR peak area ratio.

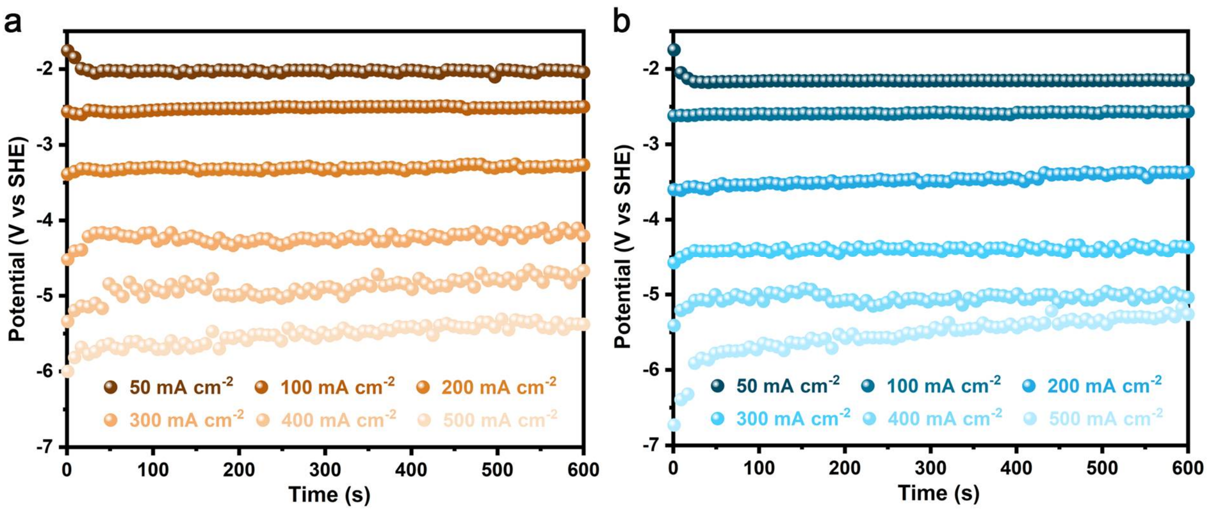


**Supplementary Figure 10. $CO_2$RR test in a flow cell.** Voltage curves for (**a**) $ZnIn_2S_4$ and (**b**) $In_2S_3$ under various current densities for 10 min in $CO_2$-fed 1M $KHCO_3$.

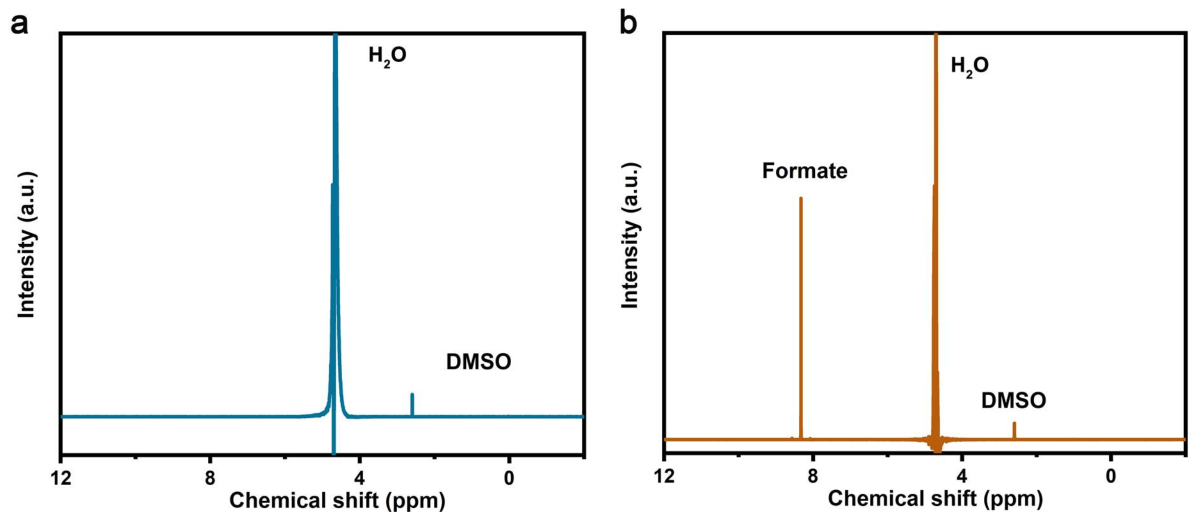


**Supplementary Figure 11. Representative $^1H$ NMR spectrums over $ZnIn_2S_4$ after electrolysis at –300 mA cm$^{-2}$ for 10 min. a,** In Ar-fed 1 M $KHCO_3$. **b,** In $CO_2$-fed 1 M $KHCO_3$. DMSO is used as an internal standard for quantification of formate. No formate is detected after electrolysis in Ar-fed 1 M $KHCO_3$, confirming that formate is formed from the $CO_2$ reduction rather than from catalysts nor carbon paper of the working electrode.

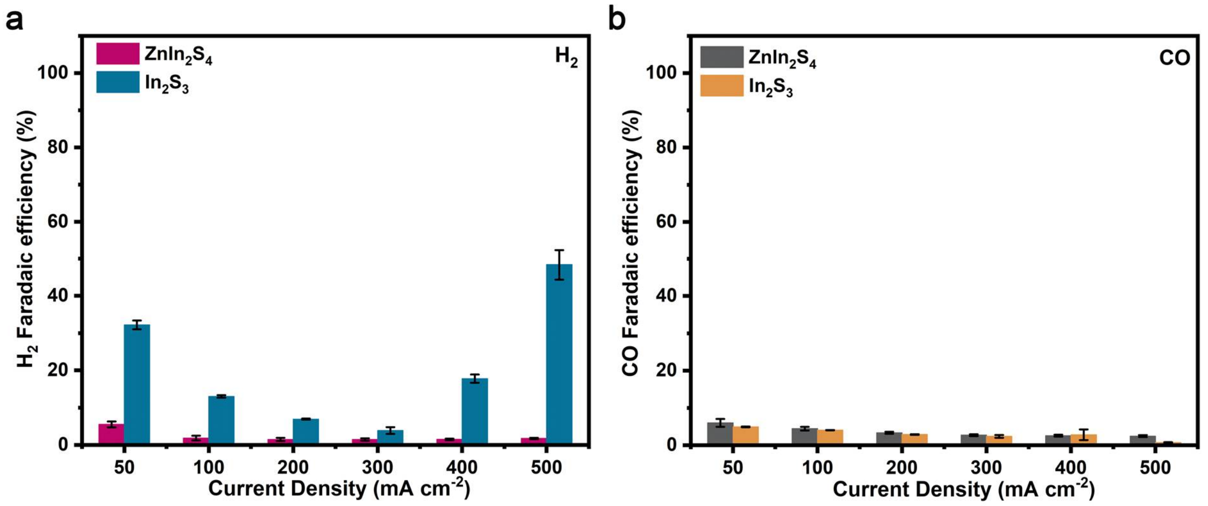


**Supplementary Figure 12. FEs for other products.** FEs for (**a**) $H_2$ and (**b**) CO on the $ZnIn_2S_4$ and $In_2S_3$ under various current densities. The error bars represent the standard deviation of three independent measurements.

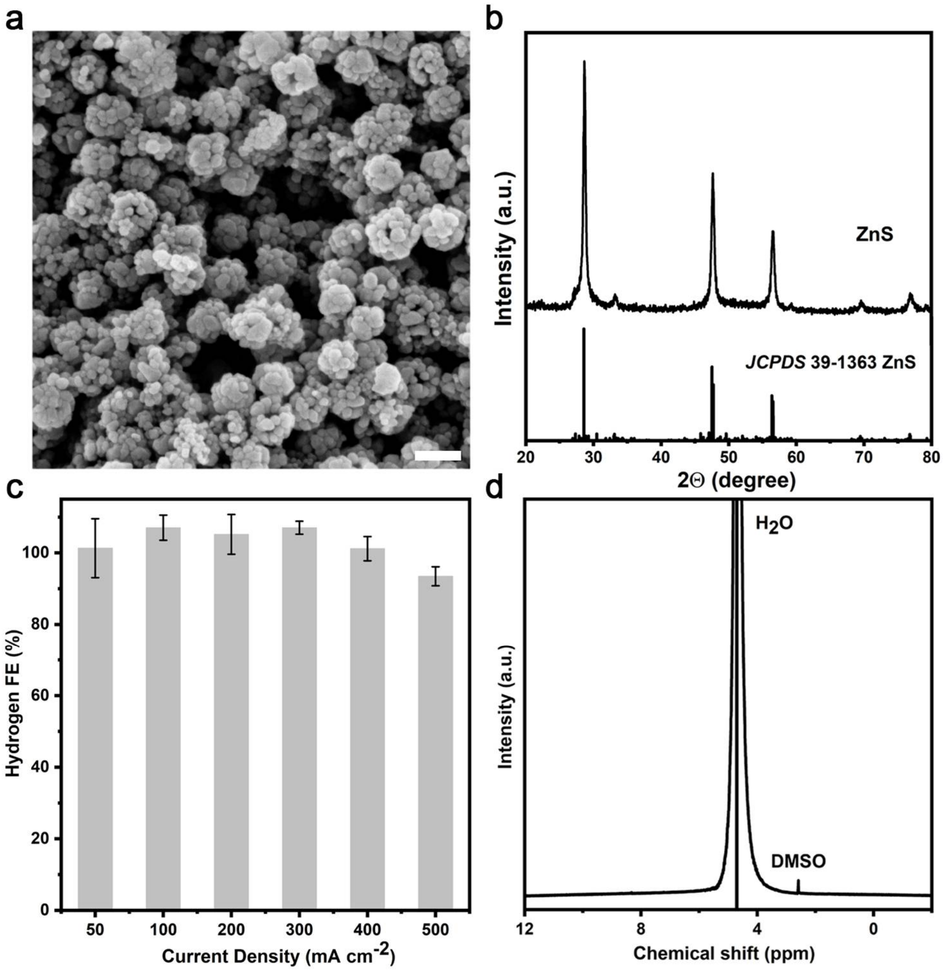


**Supplementary Figure 13. Physical characterization and $CO_2$RR performance of the ZnS. a,** SEM image. **b,** XRD pattern. **c,** $H_2$ FE of ZnS in a flow cell under various current densities. The error bars represent the standard deviation of three independent measurements. **d,** Representative $^1$H NMR spectrum over ZnS after $CO_2$ electrolysis at 300 mA cm$^{-2}$ for 10 min in $CO_2$-fed 1 M $KHCO_3$. For hexagonal ZnS, only $H_2$ was produced without any detectable carbonaceous products. Scale bar, 200 nm.

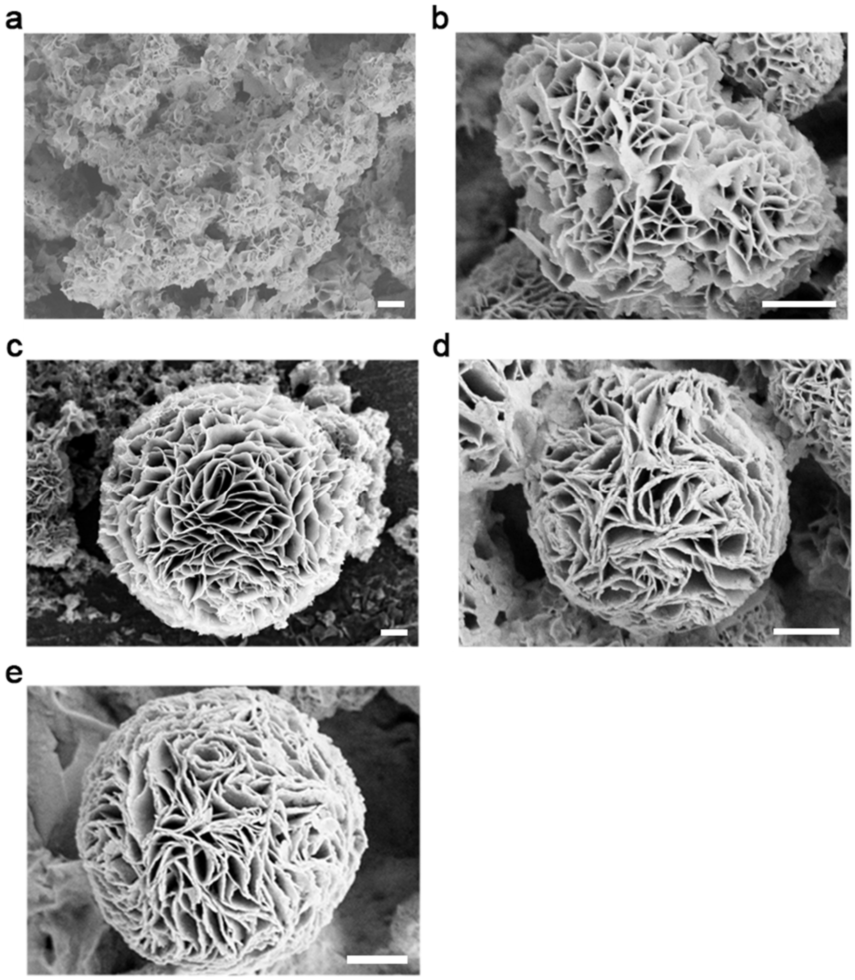


**Supplementary Figure 14. SEM images.** SEM images of the $ZnIn_2S_4$ samples obtained at 160 ºC for different reaction times: **a,** 2 h, **b,** 4 h, **c,** 6 h, **d,** 8 h, **e,** 10 h, respectively. When prolonging the reaction time, the thickness of $ZnIn_2S_4$nanosheets become thicker and rougher. Scale bars, 1 μm.

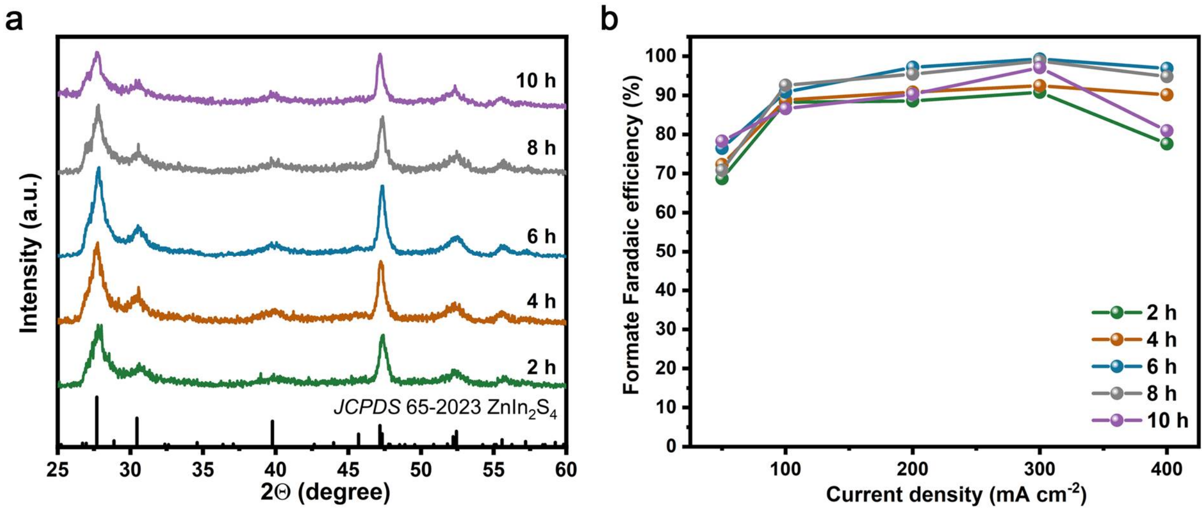


**Supplementary Figure 15. XRD pattern and $CO_2$RR performance of $ZnIn_2S_4$ samples obtained at 160 ºC for different reaction times. a,** XRD patterns. **b,** FE for formate product. The samples obtained at different reaction time are readily indexed to hexagonal $ZnIn_2S_4$ phase. We found that the thickness of nanosheets in the $ZnIn_2S_4$ microflowers shows slight effect on the $CO_2$RR properties, with the thickness of 8.686 nm obtained at 6 h showing the best performance.

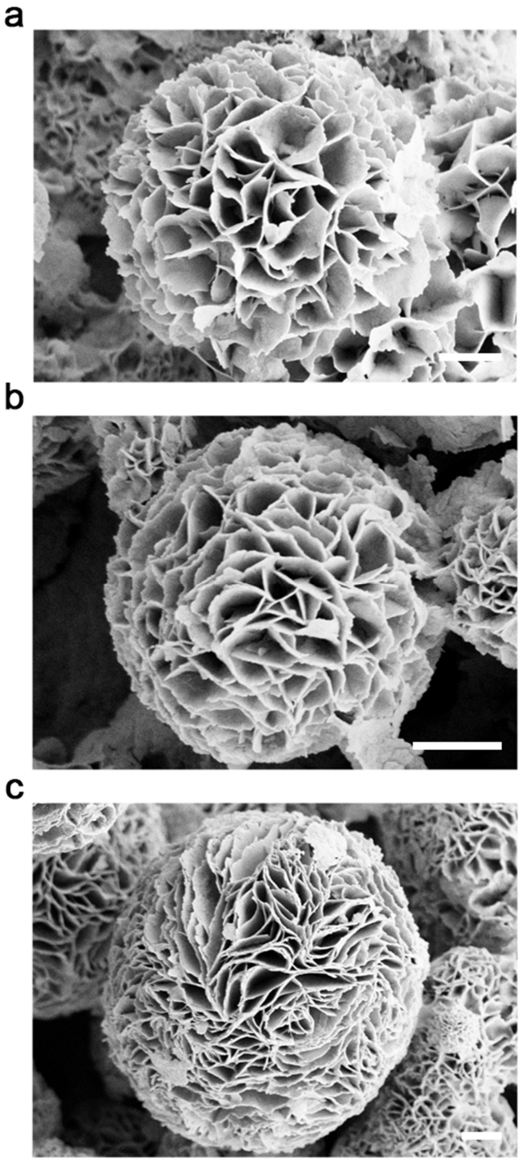

**Supplementary Figure 16. SEM images.** SEM images of $ZnIn_2S_4$ samples obtained at different reaction temperature for 6 h: **a,** 120 ºC, **b,** 140 ºC, **c,** 180 ºC, respectively. It shows that with the increase of reaction temperature, the hierarchically organized nanosheets of $ZnIn_2S_4$ microflowers become bigger and denser. Scale bars, 1 μm.

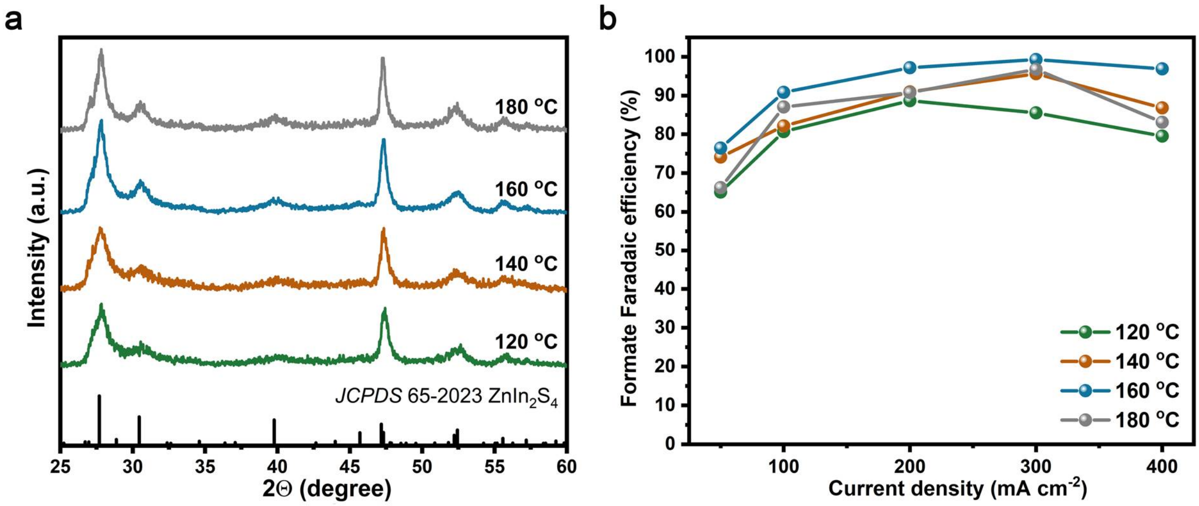


**Supplementary Figure 17. Characterization and $CO_2$RR performance of $ZnIn_2S_4$ samples obtained at different reaction temperature for 6 h. a,** XRD patterns. **b,** FE for formate product. XRD patterns confirm that all the samples obtained at different reaction temperature belong to hexagonal $ZnIn_2S_4$, and the sample obtained at 160 ºC is most capable of producing formate.

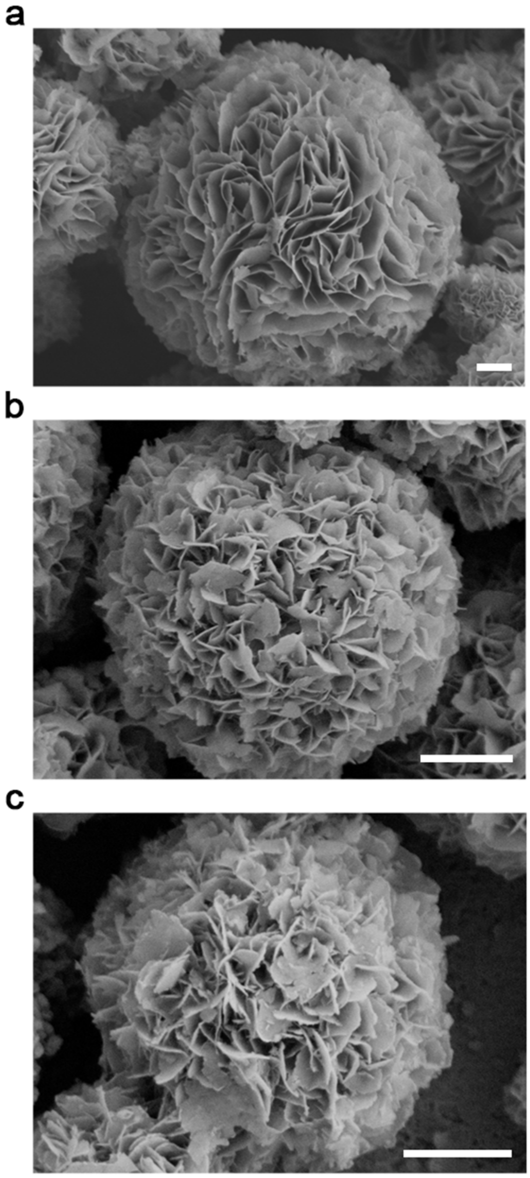


**Supplementary Figure 18. SEM images.** SEM images of $ZnIn_2S_4$ samples obtained at 160 °C for 6 h with different Zn:In ratios: **a,** 1:3, **b,** 1:1, **c,** 2:1, respectively. The ZnIn ternary sulfide with different Zn:In ratios were synthesized by adding $ZnCl_2$ and $InCl_3 \cdot 4H_2O$ in different proportions during the synthesis. The Zn:In ratios were determined by the molar ratios of added $ZnCl_2$ and $InCl_3 \cdot 4H_2O$. Scale bars, 1 μm.

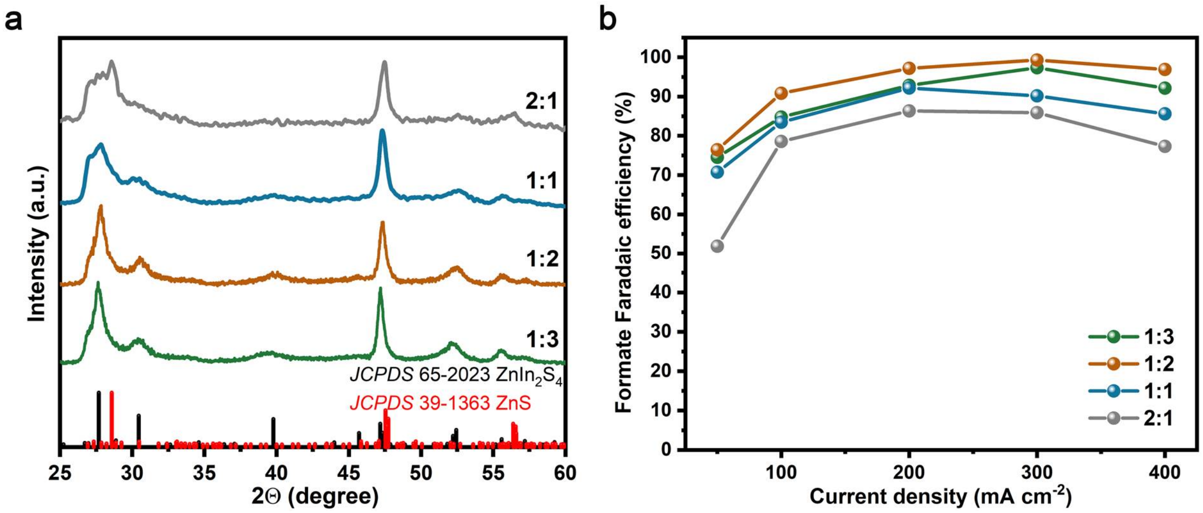


**Supplementary Figure 19. Characterization and $CO_2$RR performance of samples obtained at 160 °C for 6 h with different Zn:In ratios. a,** XRD patterns. **b,** FE for formate product. With Zn:In ratio increasing to 1:1, it appears some diffraction peaks belonging to ZnS, indicating the coexistence of $ZnIn_2S_4$ and ZnS phases. The best $CO_2$RR performance was obtained by the catalyst with the Zn:In ratio of 1:2, whereas the performance got worse with higher Zn:In ratio.

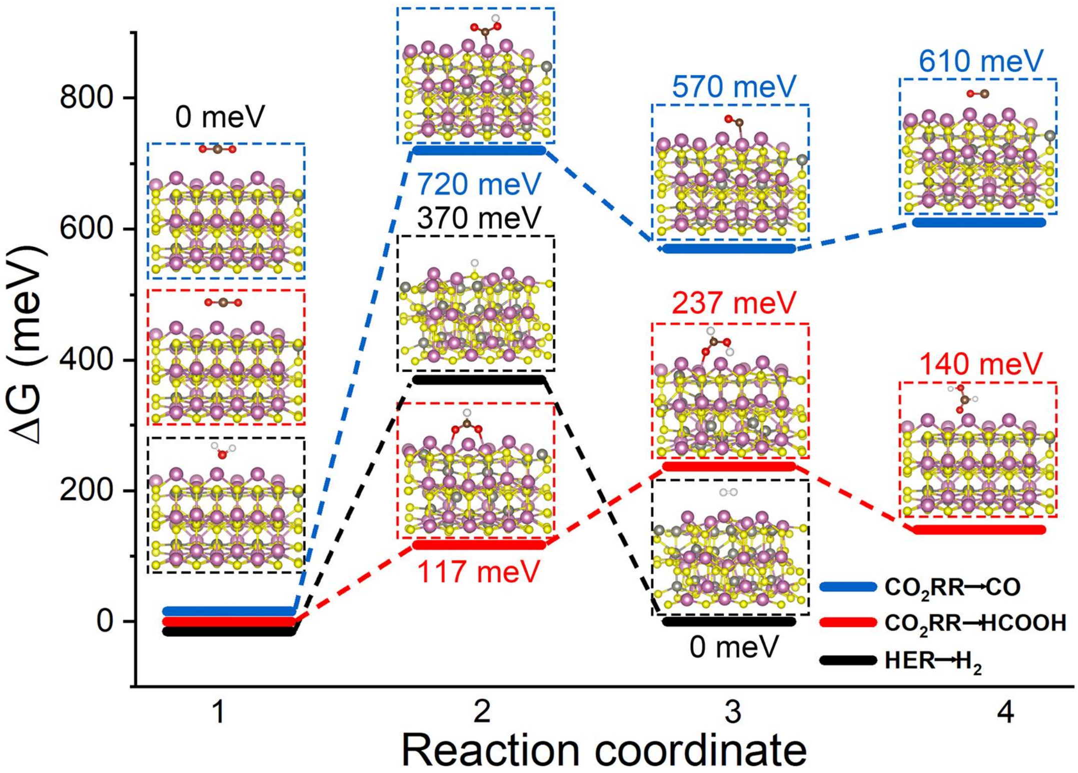


**Supplementary Figure 20. DFT calculation and reaction schemes on $ZnIn_2S_4$ (102) surface.** The blank, red and blue curves indicate the HER to $H_2$, $CO_2RR$ to HCOOH and CO pathway, respectively. The corresponding schematic reaction processes are displayed in blank, red and blue dotted boxes. The purple, yellow, gray, red, white and brown balls represent In, S, Zn, O, H and C, respectively.

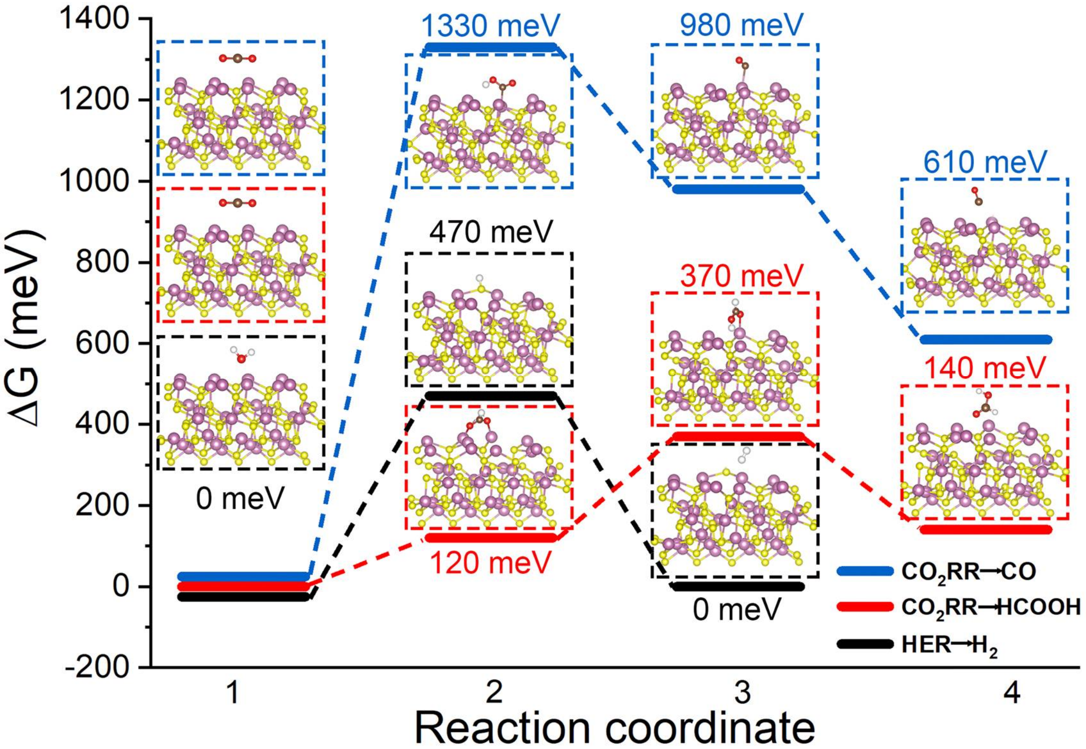


**Supplementary Figure 21. DFT calculation and reaction scheme on $In_2S_3$ (311) surface.** The blank, red and blue curves indicate the HER to $H_2$, $CO_2RR$ to HCOOH and CO pathway, respectively. The corresponding schematic reaction processes are displayed in blank, red and blue dotted boxes. The purple, yellow, red, white and brown balls represent In, S, O, H and C, respectively.

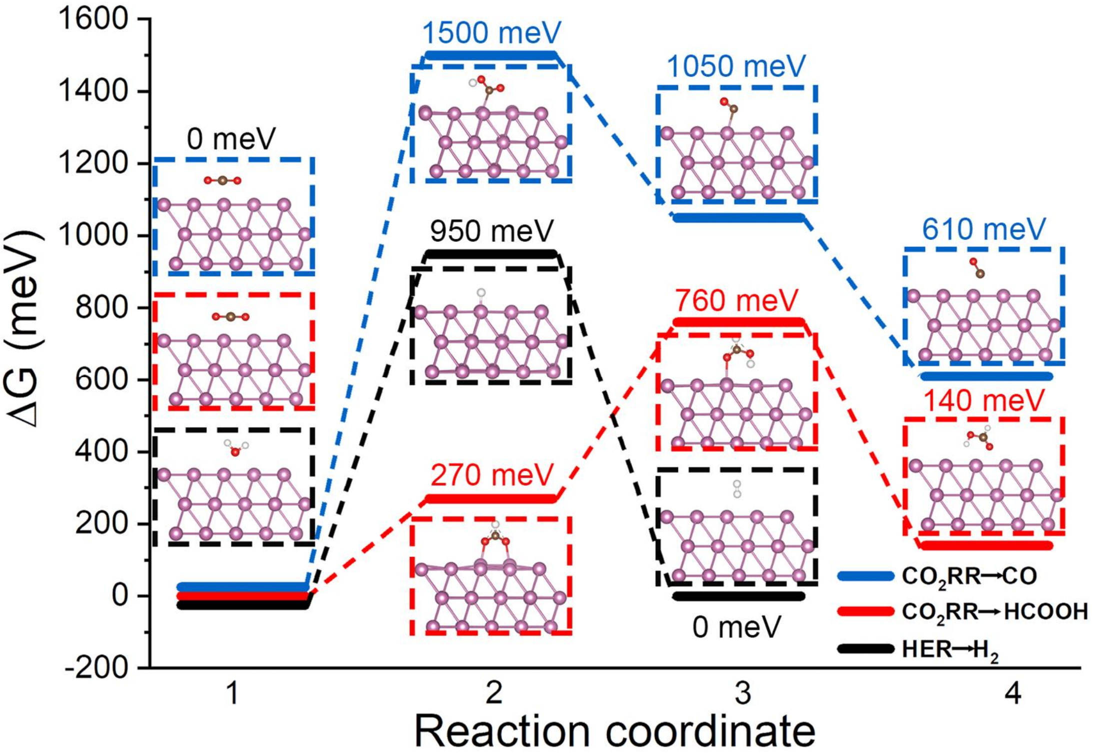


**Supplementary Figure 22. DFT calculation and reaction scheme on In (101) surface.** The blank, red and blue curves indicate the HER to $H_2$, $CO_2RR$ to HCOOH and CO pathway, respectively. The corresponding schematic reaction processes are displayed in blank, red and blue dotted boxes. The purple, red, white and brown balls represent In, O, H and C, respectively.

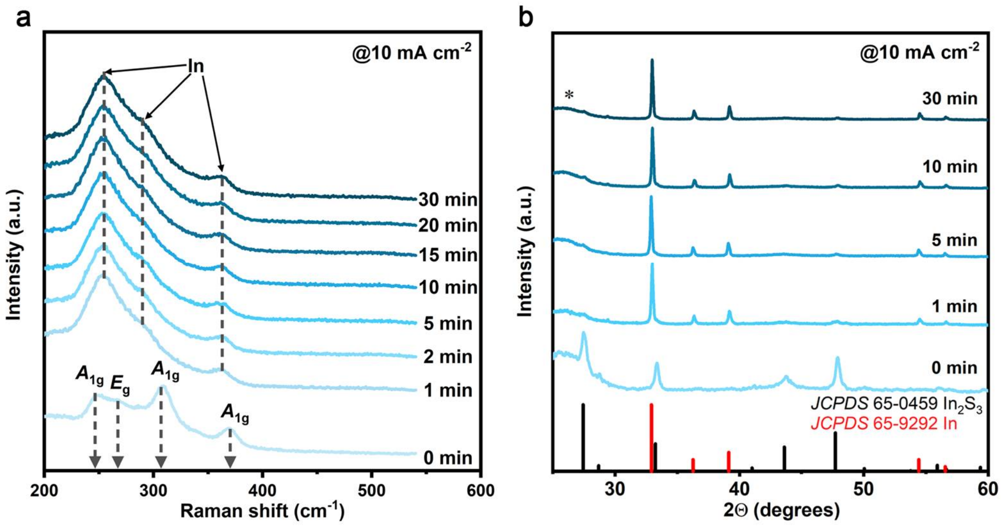


**Supplementary Figure 23. *Operando* Raman spectra and XRD patterns.** The *Operando* Raman spectra (**a**) and XRD patterns (**b**) of $In_2S_3$ under $CO_2$ electrolysis performed at 10 mA cm$^{-2}$ for different reaction times. The peaks from the substrate are labelled by black asterisk. It shows that the Raman signals of $In_2S_3$ ($A_{1g}$ and $E_g$) disappeared rapidly within 1 min, whereas In characteristic peaks appear, suggesting a quick phase transition from $In_2S_3$ to metallic In, consistent with the XRD results.

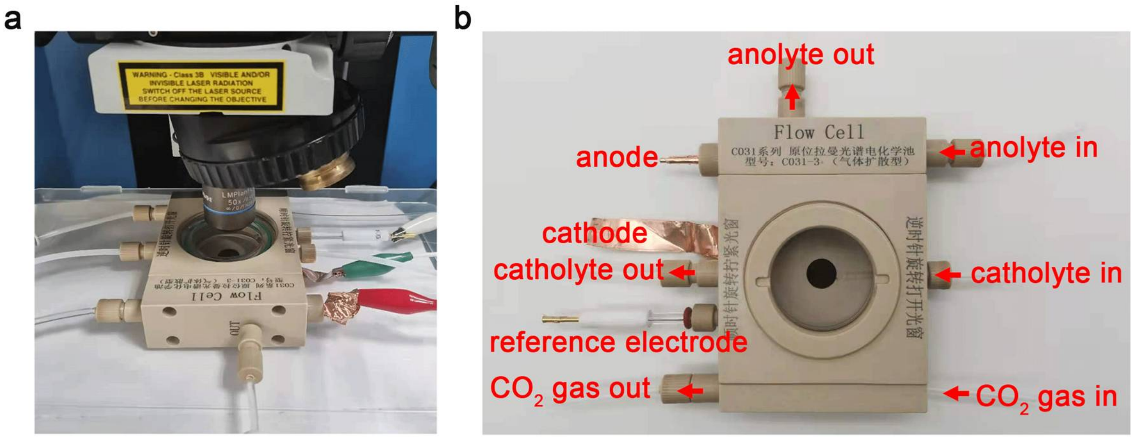


**Supplementary Figure 24. *Operando* Raman setup.** (**a**) Photograph of the *operando* Raman setup. (**b**) Photograph with notes that give detailed descriptions about the cell for *operando* Raman measurements. We took a three-electrode system to obtain *operando* Raman spectra, where the $In_2S_3$ GDE, an Ag/AgCl (saturated KCl) and Pt wire were used as work electrode, reference electrode and counter electrode, respectively. The anode and cathode chambers were separated by the cation exchange membrane (CEM). During the test, the catholyte and anolyte (1 M $KHCO_3$) flowed into the system through a peristaltic pump, and $CO_2$ gas flowed in the system without interruption. The 785 nm laser was taken as light source to shine directly on the catalyst surface. When the test starts, a computer synchronously collected the Raman signals at a cathodic current density of 10 mA $cm^{-2}$ as a function of reaction time.

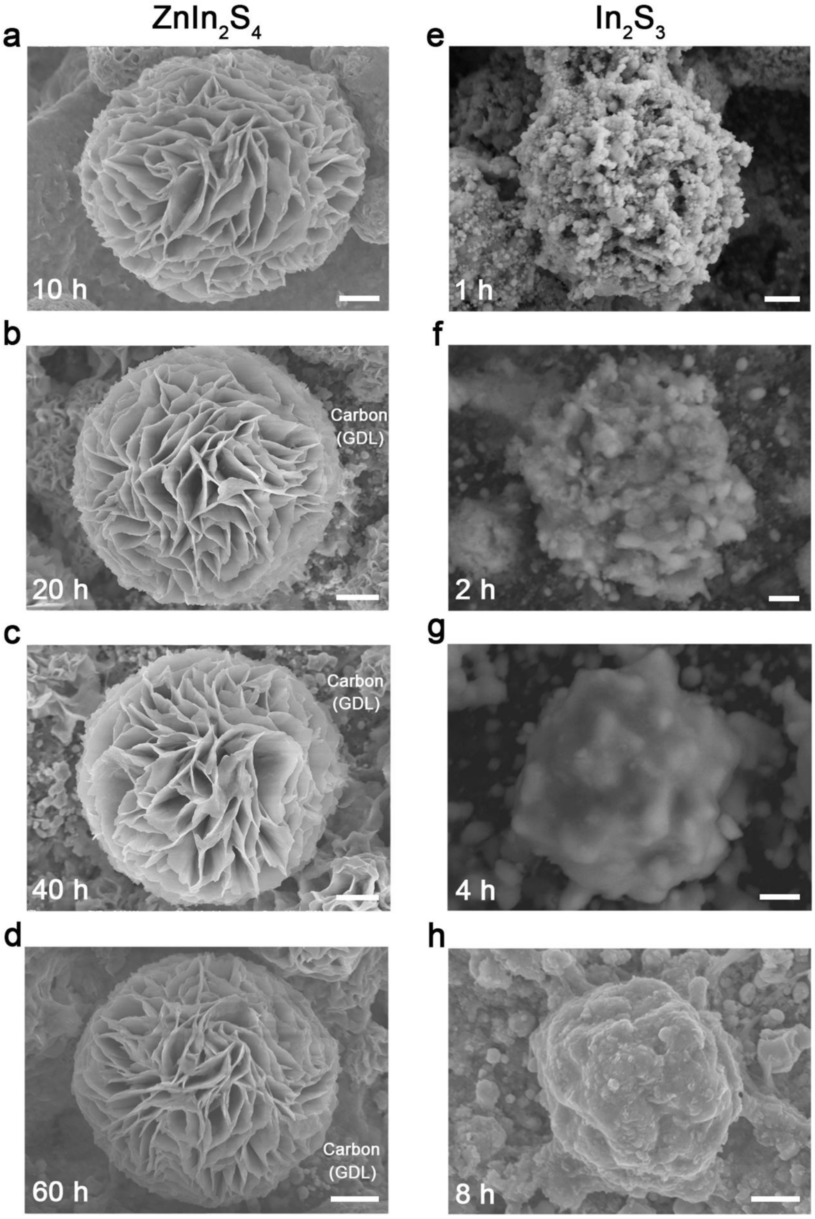


**Supplementary Figure 25. SEM images after stability test. a-d,** SEM images of the $ZnIn_2S_4$ after $CO_2RR$ at 300 mA cm$^{-2}$ for 10 h (**a**), 20 h (**b**), 40 h (**c**) and 60 h (**d**). It shows that the $ZnIn_2S_4$ can retain the original morphology even after 60 hours of operation at 300 mA cm$^{-2}$, further proving its stable morphology and structure responsible for good performance stability. **e-h,** SEM images of the $In_2S_3$ after $CO_2RR$ at 300 mA cm$^{-2}$ for 1 h (**e**), 2 h (**f**), 4 h (**g**) and 8 h (**h**). The results reveal that the morphology of $In_2S_3$ collapsed dramatically, and finally evolved into irregular particles, which is consistent well with the rapid degradation of the $CO_2RR$ performance. Scale bars: **a-d,** 1 μm, **e-h,** 500 nm.

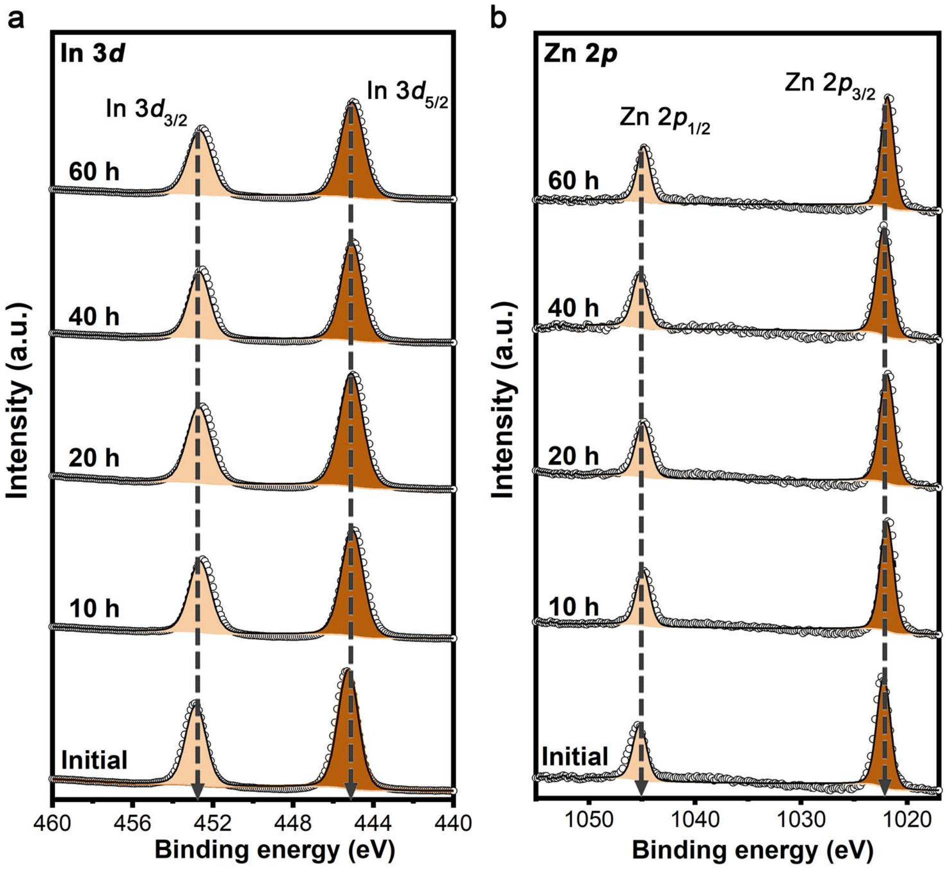


**Supplementary Figure 26. XPS spectra of $ZnIn_2S_4$.** The In 3*d* XPS spectra (**a**) and Zn 2*p* XPS spectra (**b**) of the $ZnIn_2S_4$ before and after $CO_2RR$ at 300 mA $cm^{-2}$ for 10 h, 20 h, 40 h and 60 h. The binding energy of In 3*d* and Zn 2*p* of $ZnIn_2S_4$ were retained even after 60 hours of operation, implying unchanged valance state of In and Zn.

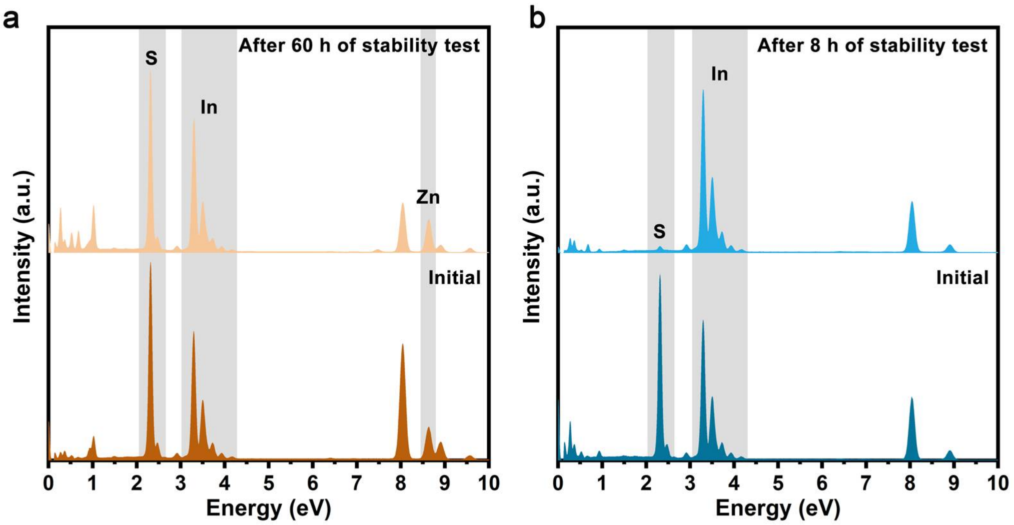


**Supplementary Figure 27. STEM-EDX. a,** The STEM-EDX of the $ZnIn_2S_4$ before and after 60 hours of operation at 300 mA $cm^{-2}$. **b,** The STEM-EDX of the $In_2S_3$ before and after 8 hours of operation at 300 mA $cm^{-2}$. The results show that the amount of S remained in $ZnIn_2S_4$ is nearly unchanged, which is starkly contrasted with $In_2S_3$ without S remained.

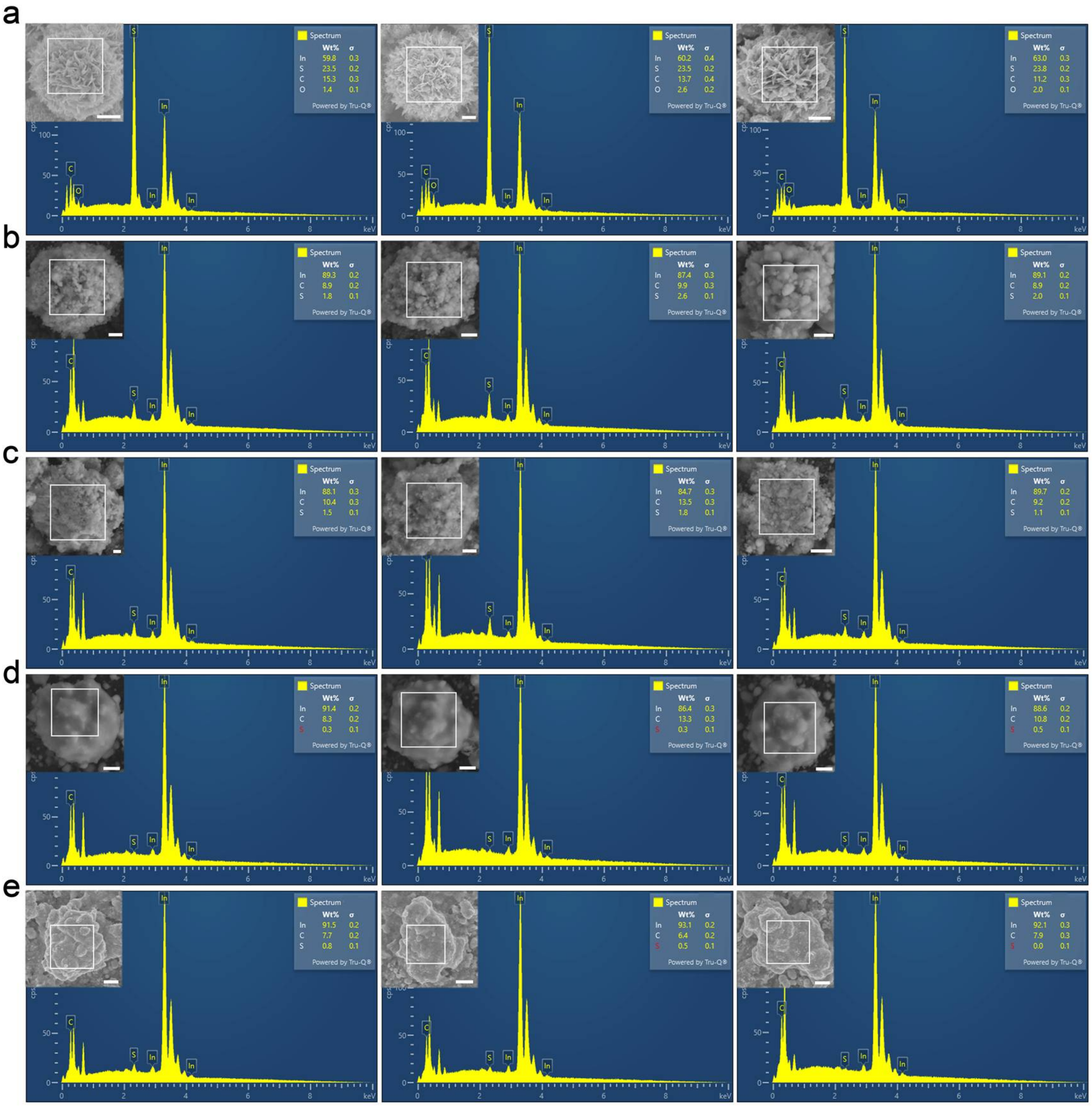


**Supplementary Figure 28. SEM-EDX on $In_2S_3$.** The SEM-EDX of the $In_2S_3$ before (**a**) and after $CO_2RR$ at 300 mA $cm^{-2}$ for 1 h (**b**), 2 h (**c**), 4 h (**d**) and 8 h (**e**). Insets are the corresponding EDX selected areas. At least three measurements were taken for separate samples and average values are represented by the standard deviation as the error bar (Fig. 3i in Main-text). Scale bars, 500 nm.

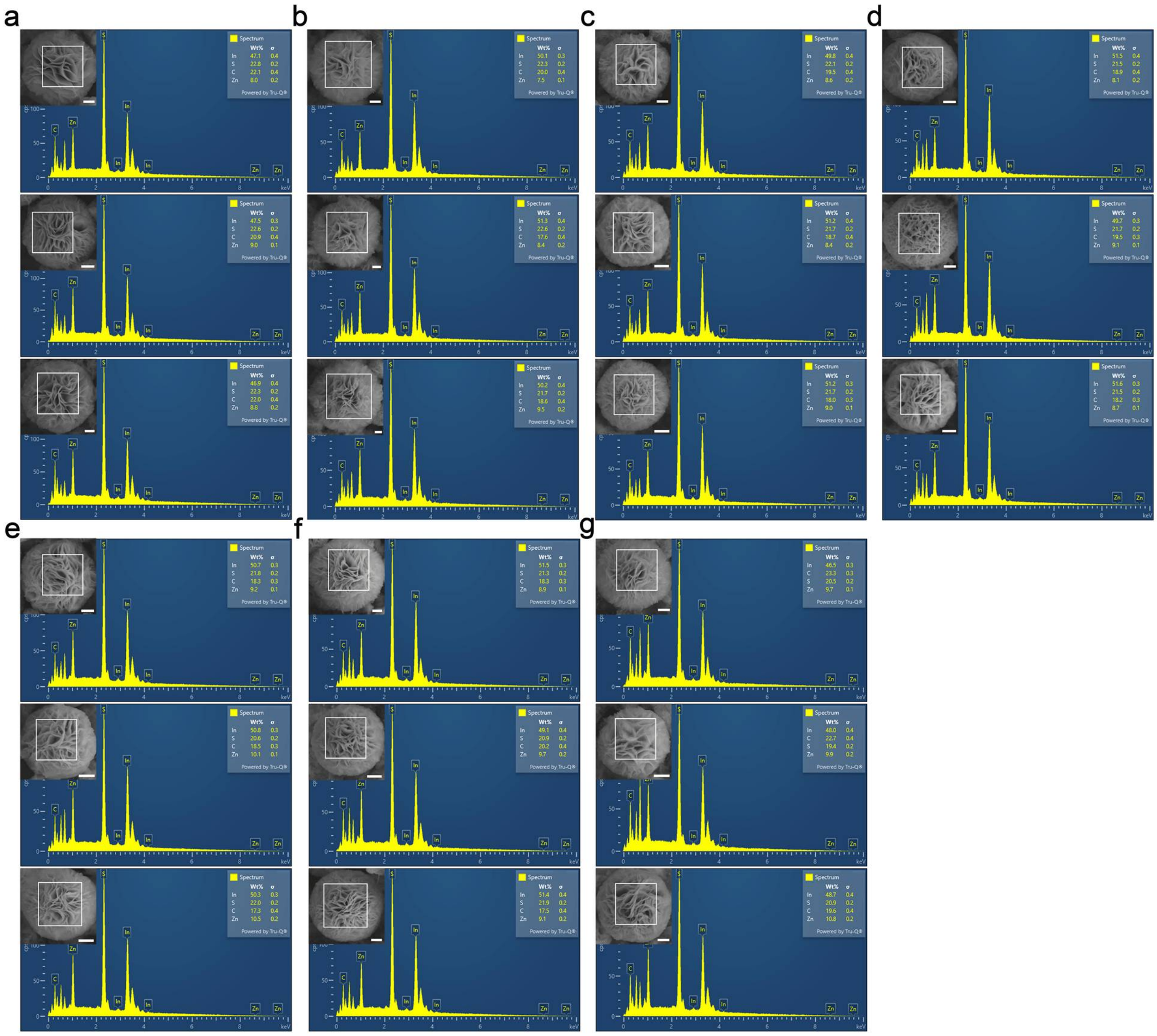


**Supplementary Figure 29. SEM-EDX on $ZnIn_2S_4$.** The SEM-EDX of the $ZnIn_2S_4$ before (**a**) and after $CO_2$RR at 300 mA $cm^{-2}$ for 1 h (**b**), 4 h (**c**), 10 h (**d**), 20 h (**e**), 40 h (**f**) and 60 h (**g**). Insets are the corresponding EDX selected areas. At least three measurements were taken for separate samples and average values are represented by the standard deviation as the error bar (Fig. 3i in Main-text). Scale bars, 1 μm.

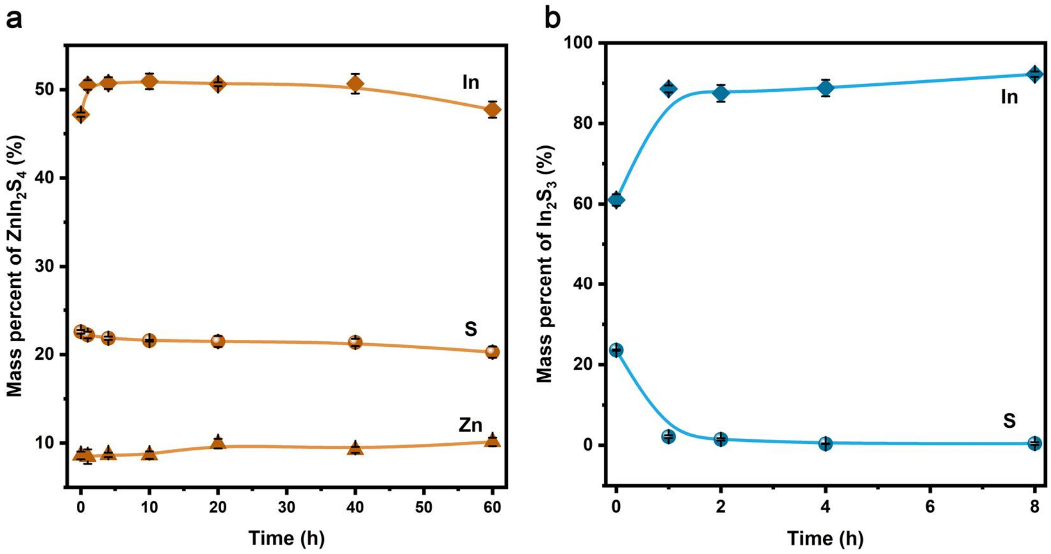


**Supplementary Figure 30. Mass percent after stability test.** The mass percent of various elements on $ZnIn_2S_4$ (**a**) and $In_2S_3$ (**b**) after $CO_2RR$ at 300 mA $cm^{-2}$ under various operating times. The error bars represent the standard deviation of three independent measurements.

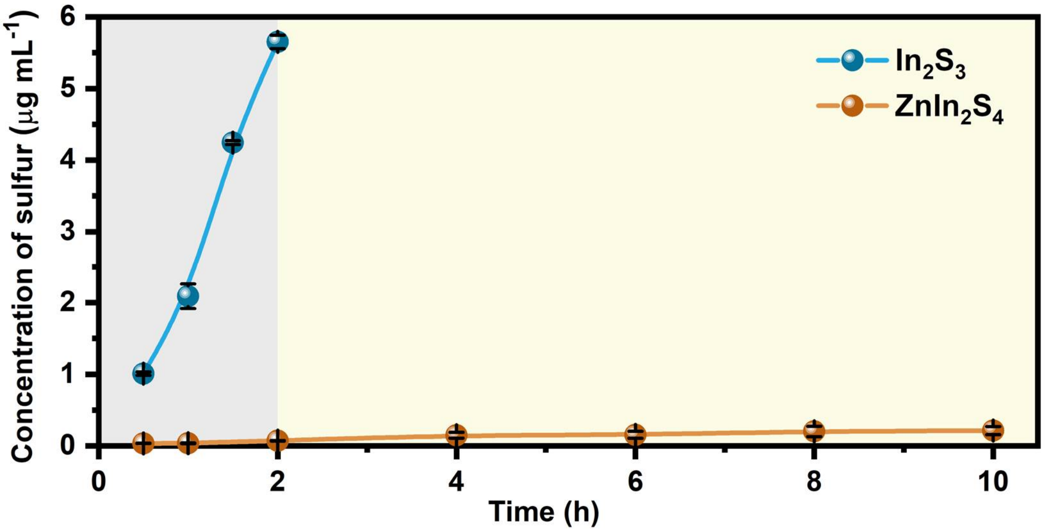


**Supplementary Figure 31. ICP-AES.** The ICP-AES measurements compare the concentration of S dissolved into 500 mL 1 M $KHCO_3$ electrolyte after $CO_2RR$ at 300 mA $cm^{-2}$ on $ZnIn_2S_4$ and $In_2S_3$. It demonstrates significantly increased S amount in electrolyte for $In_2S_3$. By stark contrast, negligible S in electrolyte was detected for $ZnIn_2S_4$. The error bars represent the standard deviation of three independent measurements.

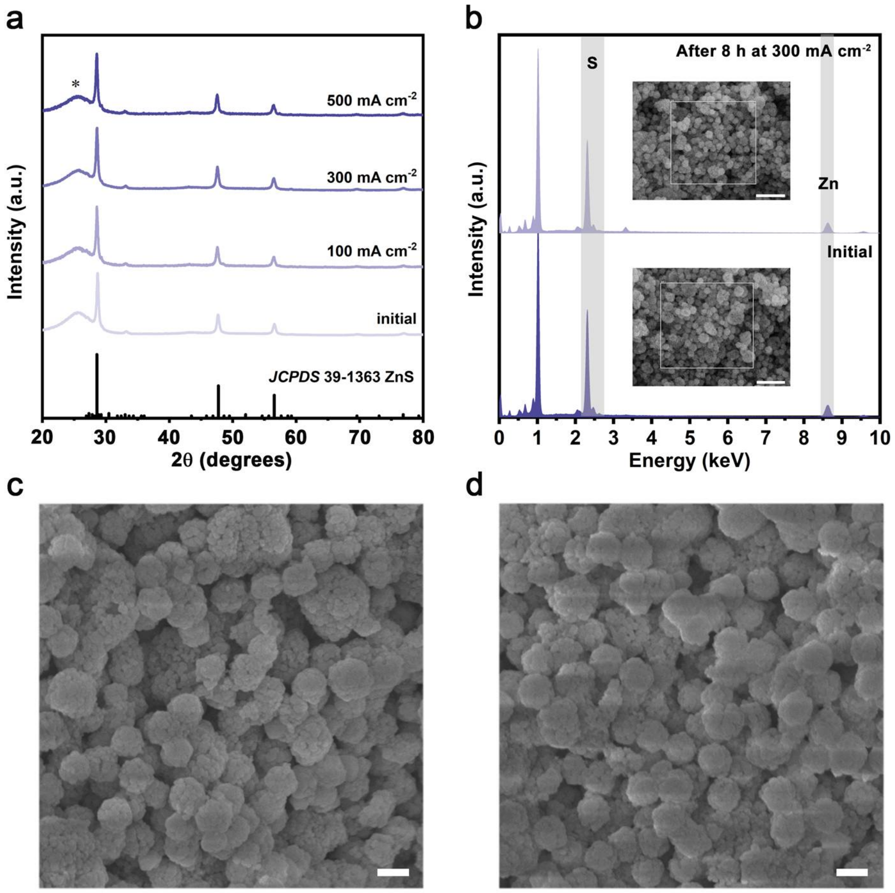


**Supplementary Figure 32. Stability evaluation of ZnS. a,** XRD patterns of ZnS after $CO_2$ electrolysis under various current densities for 10 min. The peaks from the substrate are labelled by black asterisk. **b,** SEM-EDX of ZnS before and after 8 hours of operation at 300 mA $cm^{-2}$. Insets are the corresponding EDX selected areas. Scale bars, 1 µm. **c, d,** Corresponding SEM images of ZnS before (**c**) and after (**d**) $CO_2$ electrolysis for 8 hours at 300 mA $cm^{-2}$. Scale bars, 200 nm.

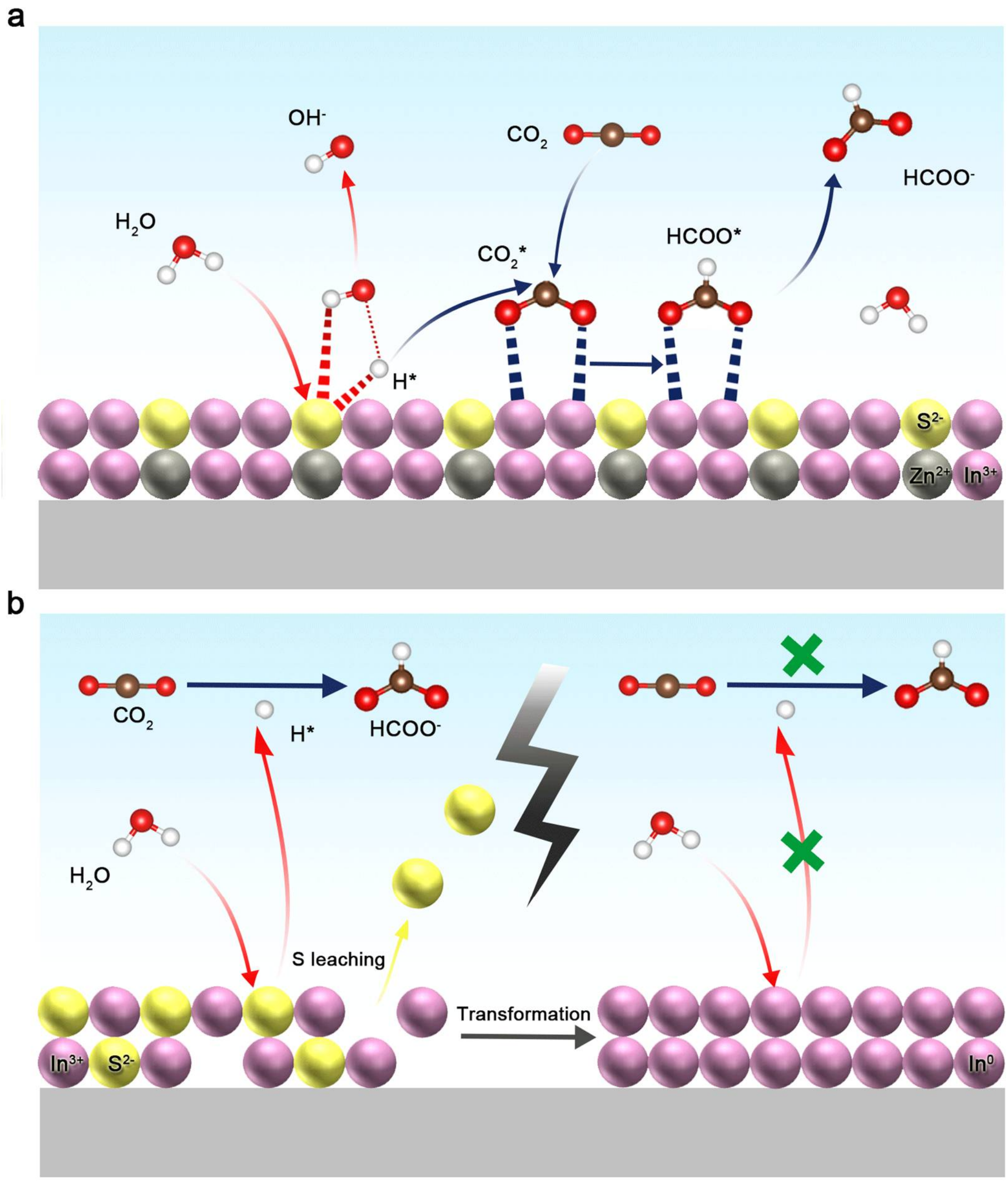


**Supplementary Figure 33. Schematic diagram for formate generation. a,** $ZnIn_2S_4$. The schematic diagram shows the effect of S in promoting water dissociation and H* formation for $CO_2$-to-formate conversion. In detail, $H_2O$ molecules on surface S sites can be activated easily to form adsorbed H* intermediate and release an $OH^-$ anion. Subsequently, the H* intermediate can combine with the adsorbed $CO_2$ to form a bound HCOO* intermediate. At last, formate is formed after HCOO* intermediate accepted an electron, and desorbs from the In active sites of $ZnIn_2S_4$. The $Zn^{2+}$ serves as an anchor to "lock" sulfur via stronger covalent interaction, thus greatly keep high activity and durability towards $CO_2$RR. **b,** $In_2S_3$. The schematic diagram shows that water dissociation readily happens on S sites of $In_2S_3$, even if not as favorable as $ZnIn_2S_4$, and then facilitates $CO_2$-to-$HCOO^-$ conversion on In sites. However, during the phase transformation from $In_2S_3$ to In, a large of S seriously dissolves into electrolyte, which resulted in very slow water dissociation and thus poor $CO_2$RR activity and stability.

**Supplementary Table 1.** Comparison of formate Faradaic efficiency, formate partial current density and formate production rate for different $CO_2RR$ catalysts in $KHCO_3$ electrolyte.

| Electrolyzer | Catalyst | Electrolyte | Formate FE (%) | $j_{Formate}$ (mA cm$^{-2}$) | Production rate (μmol cm$^{-2}$ h$^{-1}$) | Ref. |
|---|---|---|---|---|---|---|
| H-cell | In-SAs/NC | 0.5 M $KHCO_3$ | 96 | 8.52 | 158 | Angew. Chem. Int. Ed. 2020, 59, 22465 |
| | Hp-In | 0.1 M $KHCO_3$ | 90.4 | 60.75 | 1140 | J. Mater. Chem. A 2019, 7, 4505-4515 |
| | S-$In_2O_3$ | 0.5 M $KHCO_3$ | 93 | 53.1 | 1002 | Nat. Commun. 2019, 10, 892 |
| | Mn doped $In_2S_3$ | 0.1 M $KHCO_3$ | 86 | 17.3 | 322 | Nano Lett. 2019, 19, 6547−6553 |
| | In–$In_2S_3$ | 1.0 M $KHCO_3$ | 76 | 40.3 | 752 | Chem. Commun. 2020, 56, 4212-4215 |
| | $ZnInO_X$/NCF | 0.5 M $KHCO_3$ | 90.5 | 14 | 261 | Appl. Catal. B Environ. 2020, 279, 119383 |
| | $Zn_{0.95}In_{0.05}$ | 0.5 M $KHCO_3$ | 95 | 20.9 | 400 | J. Mater. Chem. A 2019, 7, 22879-22883 |
| | 5% Ni-$SnS_2$ | 0.1 M $KHCO_3$ | 79 | 15.5 | 289 | Angew. Chem. Int. Ed. 2018, 57, 10954-10958 |
| | Sn−Cu alloy | 0.1 M $KHCO_3$ | 82 | 18.9 | 352 | J. Mater. Chem. A 2019, 7, 27514-27521 |
| Flow cell | Bi@Sn | 2.0 M $KHCO_3$ | 92.2 | 230.5 | 4299 | Adv. Sci. 2020, 7, 1902989 |
| | $Bi_2O_3$ NT | 1.0 M $KHCO_3$ | 95 | 119.5 | 2229 | Nat. Commun. 2019, 10, 2807 |
| | N-Sn(S) | 0.1 M $KHCO_3$ | 93.3 | 21 | 1358 | Nano Lett. 2020, 20, 8, 6097–6103 |
| | $SnO_2$ NP | 1.0 M $KHCO_3$ | 64 | 92.8 | 2000 | J. Mater. Chem. A 2018, 6, 10313-10319 |
| | Bi-ene | 1.0 M $KHCO_3$ | 99.6 | 99.6 | 1858 | Angew. Chem. Int. Ed. 2020, 59, 15014 |
| | BiOBr | 2.0 M $KHCO_3$ | 90 | 180 | 3358 | Adv. Mater. 2018, 30, 1802858 |
| | Bi-NSs | 1.0 M $KHCO_3$ | 86.9 | 373 | 6954 | Adv. Energy Mater. 2020, 10, 2001709 |
| | S-$BiVO_4$ | 1.0 M $KHCO_3$ | 97.4 | 105.4 | 1966 | Adv. Funct. Mater. 2021, 31, 2006704 |
| | nBuLi-Bi | 1.0 M $KHCO_3$ | 92 | 460 | 8580 | Nat. Commun. 2020, 11, 3633 |
| | $ZnIn_2S_4$ | 1.0 M $KHCO_3$ | 99.3 | 297.8 | 5556 | This work |
| | | | 96.9 | 387.6 | 7229 | |
| | | | 95.4 | 476.8 | 8894 | |
